# Drugs in the GIST Field (Therapeutic Targets and Clinical Trial Staging)

**DOI:** 10.2174/1567201820666221122120657

**Published:** 2023-09-28

**Authors:** Chen Huang, Xinli Ma, Ming Wang, Hui Cao

**Affiliations:** 1 Department of Gastrointestinal Surgery, Renji Hospital, School of Medicine, Shanghai Jiao Tong University, Shanghai, China

**Keywords:** GIST, targeted therapy, therapeutic targets, clinical trials, TKI, pharmaprojects

## Abstract

**Background:**

Molecular targeted therapies are the most important type of medical treatment for GIST, but the development of GIST drugs and their targets have not been summarized.

**Methods:**

Drugs in the field of GIST were analyzed and collated through Pharmaprojects, ClinicalTrials.gov and PharmaGO databases.

**Results:**

As of 2021, there are 75 drugs that have appeared in the GIST clinical trials. The six most frequent targets in GIST clinical trials, in descending order of frequency, were KIT, PDGFRA, KDR (VEGFR2), FLT3, FLT1 (VEGFR1), and FLT4/VEGFR3. Only 8 drugs are in preclinical research. There are challenges in the development of new drugs for GIST.

**Conclusion:**

This article analyzes and summarizes the general situation of GIST drugs, the target distribution of GIST drugs, and the trends in GIST drug-related clinical trials.

## 1 INTRODUCTION

GIST (Gastrointestinal stromal tumors) are the most common type of sarcoma. Most GIST have typical activating oncogene mutation profiles. The vast majority have mutations in KIT, followed by PDGFRA, with some other rare mutations including those in SDH, NF1, BRAF and NTRK3 [1-4]. Cytotoxic treatments are not suitable for GIST treatment [5]. Molecular targeted therapies are the most important part of the medical treatment of GIST [3, 6].

## 2 MATERIALS AND METHODS

### 2.1 Pharmaprojects

The vast majority of the drug data (therapeutic targets and development status) for this study were obtained from the Pharmaprojects database (https://citeline.informa.com/).

### 2.2 ClinicalTrials.gov

All data from clinical trials involved in this study were obtained from the ClinicalTrials.gov database (https://clinicaltrials.gov/).

### 2.3 PharmaGO

The drug data for this study were also supplemented by the PharmaGO database (https://db.pharmcube.com/, PHARMCUBE).

### 2.4 Statistical Analysis

Excel 2013, R version 4.2.0 and GraphPad 7.0 software were used for performing the statistical analyses and data visualization in this study.

## 3 RESULTS

### 3.1 Overview of Drugs in Clinical Trials Related to GIST

As of 2021, there are 75 drugs have appeared in the GIST clinical trials. 5 drugs (imatinib mesilate, sunitinib, regorafenib, ripretinib and avapritinib) have been approved for marketing, of which imatinib and sunitinib have already been launched widely. All 5 drugs above are tyrosine kinase inhibitors (TKIs). Masitinib (AB-1010) once made it to phase III clinical trial and even pre-registration but is currently ceased. There are 9 other drugs that have entered phase 3 clinical studies, 5 of which have the opportunity to enter the clinical treatment of GIST (in phase III clinical trials and the development status is active), namely, bevacizumab, crenolanib, famitinib, nilotinib and pimitespib (Table **1**).

22 drugs entered phase II clinical trials, of which 16 had an active status. 23 entered phase I clinical trials, of which 16 had an active status. 15 entered preclinical status, of which only 8 had an active status. (Table **S1**, full list of drugs in the GIST field and their development status.)

### 3.2 Targets of Launched Drugs [Non-TKI Drugs will be Marked with an * in the Upper Right Corner]

#### 3.2.1 Imatinib Mesilate

Imatinib mesilate (Gleevec; Glivec; imatinib; Ruvise; CGP-57148B; QTI-571; ST-571; STI-571; STI-571A) is a 2-phenylaminopyrimidine derivative TKI. It is known as a selective inhibitor of certain protein tyrosine kinases: KIT [stem-cell factor (SCF) receptor], PDGFRA (platelet derived growth factor receptors alpha), ABL1 (ABL proto-oncogene 1) and BCR activator of RhoGEF (Rho family guanine nucleotide exchange factor) + GTPase (guanosine triphosphatase) [7-10] and is produced by the Novartis (Table **2**).

#### 3.2.2 Sunitinib

Sunitinib (sunitinib malate; Sutene; Sutent; PHA-290940AD; SU-010398; SU-011248; SU-11248; SUO11248) is an oxindole TKI. Sunitinib has been identified as an inhibitor of PDGFRA, FLT3 [FLT (fms-related tyrosine kinase)], KIT, RET (glial cell-line derived neurotrophic factor receptor), FLT1 (VEGFR1) [VEGFR (vascular endothelial growth factor receptors)], KDR (kinase insert domain receptor, VEGFR2) and FLT4 (VEGFR3) [11-15] and is produced by the Pfizer (Table **2**).

#### 3.2.3 Regorafenib

Regorafenib (DAST; DAST-Inhibitor; Stivarga; BAY-73-4506) is a diphenylurea TKI. Regorafenib has been identified as an inhibitor of FLT1 (VEGFR1), KDR (VEGFR2), FLT4 (VEGFR3), KIT, RET, PDGFRA, PDGFR-β (platelet derived growth factor receptors beta), B-Raf, Raf-1, FGFR1 [FGFR (fibroblast growth factor receptor)] and Tie2 [16-19] and is produced by the Bayer/Amgen (Table **2**).

#### 3.2.4 Ripretinib

Ripretinib (Qinlock; DCC-2618) is a kinase inhibitor of KIT and PDGFRA. It exerts its function by locking these kinases’ activation loops in an inactive conformation. This drug was the first known “switch-controlled” kinase inhibitor [15, 20-22] and is produced by the Deciphera Pharmaceuticals/Zai Lab (Table **2**).

#### 3.2.5 Avapritinib

Avapritinib (Ayavkit; Ayvakit; Ayvakyt; BLU-285; CS-3007) is also a “switch-controlled” kinase inhibitor targeted in unique KIT and PDGFRA activation loop mutations (KIT D816V, PDGFRA D842V mutant) [4, 19, 21, 23] and is produced by the Blueprint Medicines/CStone Pharmaceuticals (Table **2**).

### 3.3 Targets of Drugs in Active Phase III Clinical Trials

#### 3.3.1 Bevacizumab

Bevacizumab (Avastin, Altuzan, anti-VEGF MAb, bevacizumab, R-435, Ro-4876646) is an angiogenesis inhibitor targeted to VEGF (vascular endothelial growth factor A) [24-26] and is produced by the Roche (Table **3**).

#### 3.3.2 Crenolanib

Crenolanib (CP-868596, 596-26, ARO-002, crenolanib besylate, plarotinib) is a TKI targeted to PDGFR-β, PDGFRA and FLT3. Specifically, it is more selective for PDGFR than for other kinases [18, 27, 28] and is produced by Arog Pharmaceuticals/JI Shanghai Biotechnology/Astellas Pharma/Pfizer (Table **3**).

#### 3.3.3 Famitinib

Famitinib (famitinib malate, SHR-1020) is a TKI targeted to KDR (VEGFR2), FLT4 (VEGFR3), KIT and FLT3 [29, 30] and is produced by the Jiangsu Hengrui Pharmaceuticals (Table **3**).

#### 3.3.4 Nilotinib

Nilotinib (AMN-107, Tasigna) is a TKI targeting the BCR activator of RhoGEF and GTPase, ABL1, KIT and PDGFRA [31-34] and is produced by Novartis (Table **3**).

#### 3.3.5 Pimitespib*

Pimitespib (TAS-116) is a highly selective inhibitor targeted to Hsp90 (heat shock protein 90) [35, 36]. In addition, it is produced by Otsuka Holdings (Table **3**).

### 3.4 Targets of Drugs in Active Phase II Clinical Trials

#### 3.4.1 Amcasertib*

Amcasertib (BB-503, BBI-503, GB-103, GH-509) is a cancer stemness kinase inhibitor (details can be found in NCT02232620) and is produced by the 1Globe Biomedical/Sumitomo Dainippon Pharma (Table **4**).

#### 3.4.2 Anlotinib Hydrochloric

Anlotinib hydrochloric (AL-3818, ALTN, anlotinib, anlotinib hydrochloride, Focus V) is a TKI targeted to KDR (VEGFR2), FLT4 (VEGFR3), FGFR1, FGFR2, FGFR3 and FGFR4 and is produced by the Sino Biopharmaceutical [37, 38] (Table **4**).

#### 3.4.3 Apatinib

Apatinib (Aitan, apatinib mesylate, Rivoceranib, rivoceranib mesylate, YN-968D1) is a TKI targeting KDR (VEGFR2) and RET [39, 40] and is produced by the HLB LifeScience/Bukwang Pharmaceutical/Jiangsu Hengrui Pharmaceuticals (Table **4**).

#### 3.4.4 Bezuclastinib

Bezuclastinib (CGT-9486, PLX-9486) is a TKI targeting KIT [41, 42] and is produced by Cogent Biosciences/Daiichi Sankyo (Table **4**).

#### 3.4.5 Dabrafenib

Dabrafenib (Tafinlar, dabrafenib mesylate, 2118436, DRB-436, GSK-2118436) is a TKI targeting B-Raf [43, 44] and is produced by Novartis (Table **4**).

#### 3.4.6 Dovitinib Lactate

Dovitinib lactate (CHIR-258, dovitinib, GFKI-258, TKI-258, TKI-258A) is a TKI targeting FGFR1, FGFR3, FLT1 (VEGFR1), KDR (VEGFR2), FLT4 (VEGFR3), PDGFR-β, KIT, FLT3 and EGFR (epidermal growth factor receptor) [45, 46]. In addition, it is produced by Allarity Therapeutics/Novartis (Table **4**).

#### 3.4.7 Everolimus*

Everolimus (Afinitor, Afinitor Disperz, Certican, RAD, RAD-001, SDZ-RAD, Votubia, Zortress) is an mTOR (mechanistic target of rapamycin kinase) inhibitor [47, 48] and is produced by Novartis (Table **4**).

#### 3.4.8 Evofosfamide*

Evofosfamide (anticancer prodrug, Threshold, evofosfamide, TH-302) is a HAP (hypoxia-activated prodrug) [49, 50] and is produced by Molecular Templates (Table **4**).

#### 3.4.9 Ilixadencel*

Ilixadencel (allogeneic dendritic cell, Immunicum, Combig-DC, Intuvac, Intuvax) is a cell-based immune primer injected intratumorally [51, 52] and is produced by the Immunicum (Table **4**).

#### 3.4.10 Midostaurin

Midostaurin (CGP-41251, midostaurin, N-benzoyl-staurosporine, PKC-412, Rydapt) is a TKI targeted to PKC-alpha (protein kinase C alpha), FLT3, KIT, VEGF, cyclin B1, FLT1 (VEGFR1), KDR (VEGFR2) and PDGFR-β [53, 54] and is produced by Novartis (Table **4**).

#### 3.4.11 Olaratumab

Olaratumab (IMC-3G3, Lartruvo, LY-3012207) is a TKI targeting PDGFRA [55, 56]. In addition, it is produced by Bristol-Myers Squibb/Eli Lilly (Table **4**).

#### 3.4.12 Onalespib

Onalespib (AT-13387, AT13387, Hsp90 inhibitor, Astex Pharmaceuticals) is a selective inhibitor targeted to Hsp90 [57, 58]. In addition, it is produced by Otsuka Holdings (Table **4**).

#### 3.4.13 Pexidartinib

Pexidartinib (Plexxikon, PLX-3397, Turalio) is a TKI targeting KIT, CSF1 receptor (colony stimulating factor 1 receptor) receptor and FLT3 [59, 60] and is produced by the Daiichi Sankyo (Table **4**).

#### 3.4.14 Ponatinib

Ponatinib (AP-24534, Iclusig, INCB-84344, ponatinib hydrochloride) is a TKI targeted to the BCR activator of RhoGEF + GTPase, ABL, FLT1 (VEGFR1), FGFR 1, FGFR 2, FGFR 3, FGFR 4, FLT3 and TEK [34, 61] and is produced by Takeda (Table **4**).

#### 3.4.15 Trametinib*

Trametinib (1120212, GSK-1120212, GSK-1120212B, GSK-212, JTP-74057, Mekinist, Mekinist POS, TMT-212, trametinib dimethyl sulfoxide) is a MEK (mitogen-activated protein kinase kinase) inhibitor targeted to MEK1 and MEK2 [62, 63] and is produced by Novartis (Table **4**).

#### 3.4.16 Temozolomide*

Temozolomide (TEMODAR, TMZ, CCRG 81045, M&B 39831, NSC 362856) is a cytotoxic chemotherapy drug and is produced by Merck Sharp Dohme (Table **4**).

### 3.5 Active Drugs in Phase I Clinical Trials and Preclinical Research

Because phase I clinical trials and preclinical research do not address drug treatment effects, only a brief statement of relevant drugs is presented. In phase I clinical trials, 177Lu-NEOBOMB1, alpelisib, buparlisib, cabozantinib, copanlisib, dasatinib, DS-6157, lenalidomide, pazopanib, pegargiminase, plinabulin, quizartinib dihydrochloride, sapacitabine, surufatinib, tidutamab and umbralisib are active. In preclinical research, anagrelide, cediranib, GT-1708F, IM-24, NN-3201, plocabulin, sugemalimab and THE-630 are active (Table **5**).

### 3.6 Ceased Drugs

Masitinib, motesanib diphosphate, retaspimycin, ridaforolimus and trebananib were ceased at phase III. Amonafide dihydrochloride, amuvatinib hydrochloride, CNF-2024, ganetespib, NRC-AN-019 and SMi-BX1 were ceased at phase II. KTN-0158, LOP-628, MK-1496, OPB-51602, perifosine, refametinib and XL-820 were ceased at phase I. AB-515, AZD-3229, c-kit inhibitors (Deciphera), gastrointestinal stromal tumor therapy (Array BioPharma), HYGT-110, LWEL-1808 and MAAC-003 were ceased at the preclinical phase. Their production companies and therapeutic targets can be found in Table **S2**.

### 3.7 Frequency of Therapeutic Targets in GIST Clinical Trials of Medicines

**Fig. (1):**
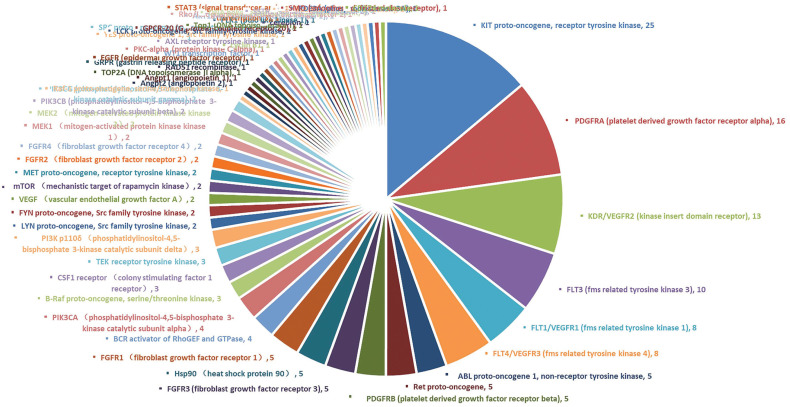
The frequency of drug targets appearing in gastrointestinal stromal tumors clinical trials.

57 targets have been involved in GIST clinical trials of medicines. The top six targets with the highest frequencies were KIT, PDGFRA, KDR (VEGFR2), FLT3, FLT1 (VEGFR1) and FLT4/VEGFR3. Of these, KIT appeared 25 times, PDGFRA appeared 16 times and KDR/VEGFR2 appeared 13 times. Targets that occurred 5-10 times are as follows: FLT3, FLT1/VEGFR1, FLT4/VEGFR3, ABL, RET, PDGFRB, FGFR3, Hsp90 and FGFR1. Targets that occurred 2-4 times are as follows: BCR activator of RhoGEF + GTPase, PIK3CA, B-Raf, CSF1 receptor (colony stimulating factor 1 receptor), TEK, PI3K p110δ, LYN, FYN, VEGF, mTOR, MET, FGFR2, FGFR4, MEK1, MEK2, PIK3CB and PIK3CG (Fig. **1**). The remaining targets only appeared once and the data are shown in Table **S3**.

### 3.8 GIST Trials

**Fig. (2):**
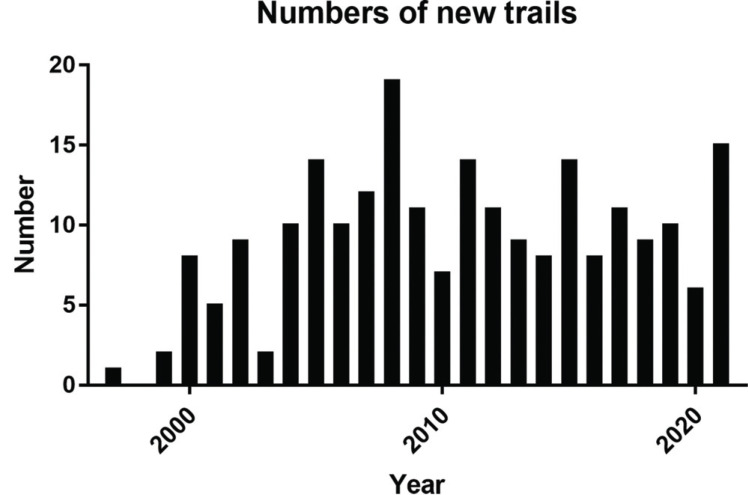
Numbers of new trials for drugs for gastrointestinal stromal tumors.

As of 2021, there were 225 clinical trials in the field of GIST drug therapy. The first clinical trial of medicines related to GIST began in 1997. The number increased significantly from 1997 to 2008 and remained stable from 2008 to 2021 (Fig. **2**). Combined with the previous findings, the number of preclinical studies of GIST is low. Therefore, we did not observe a significant growth trend in new drugs for the treatment of GIST.

## 4 DISCUSSION

This article has clear clinical value for the use of drugs in the GIST field. To the best of our knowledge, this is the first article to showcase all GIST-related drugs and their progress to the market. We have carefully compiled the aliases of the drugs, their targets, and the manufacturing companies. For researchers, this paper reduces the unnecessary workload in retrieval. For patients who have failed all 4 lines of therapy and their supervising physicians, a reliable and clear range of drug options is provided by describing the highest progress of the drug to market.

After obtaining an up-to-date tissue specimen (surgery or puncture biopsy) from the patient, physicians can perform next-generation sequencing and discover mutated genes of the tumor. Mutated genes mean that they are potential targets for treatment. In this paper, it is possible to quickly find the most reliable drugs for the corresponding therapeutic targets and to develop new treatment regimens.

The most successful GIST drugs have been demonstrated in the latest guidelines. According to the latest guideline (nccn guidelines version 2.2022 GIST), imatinib and avapritinib can be used as neoadjuvant therapy. For adjuvant therapy of resectable GIST, imatinib is the only choice. The following drugs are used for systemic treatment of unresectable, progressive or metastatic GIST. Imatinib, avapritinib, larotrectinib and entrectinib were placed in first-line therapy, sunitinib and dasatinib were used as second-line therapy, and regorafenib and ripretinib were respectively used as third- and fourth-line therapy.

Notably, GIST treatment is also available with approved “pan-tumor” drugs. Larotrectinib and entrectinib are two TKI drugs that have been approved for use in solid tumors harboring NTRK fusions. The indication of these two drugs did not mention “GIST”, so we did not consider larotrectinib and entrectinib in this article. Here, we would like to remind our readers of this fact.

In the future, matching drug targets with artificial intelligence may be performed. The strategy of using mutation targets to find drugs and using drug targets to find applicable diseases is very mature. However, in other words, it is also very old-fashioned. GIST are tumorigenic tumors with relatively simple causes and pathways, and we hope there will be a breakthrough in GIST drug development by using machine learning or artificial intelligence.

In clinical work, surgeons and medical oncologists dealing with GIST patients must consider the socioeconomic situation of the patients. GIST became available for drug treatment only after 2000 when imatinib was known as an expensive drug in China. GIST require long-term disease control, so they impose a major financial burden on the patient's family. We have witnessed a large number of patients who have given up their medication for financial reasons. To date, GIST drug therapy has started with little financial pressure, and the price of imatinib has decreased. However, newer targeted drugs are still selling at high prices due to their high development costs. Therefore, we call on the doctors who treat GIST to care for their patients in addition to their disease.

## CONCLUSION

To analyze and summarize the overall situation of GIST drugs by the end of 2021, this study used Pharmaprojects, ClinicalTrials.gov and PharmaGO databases. The conclusions obtained had 75 drugs appearing in GIST clinical trials of drugs. The six most frequent targets in GIST clinical trials were KIT, PDGFRA, KDR (VEGFR2), FLT3, FLT1 (VEGFR1), and FLT4/VEGFR3 in that order.

There are challenges in the development of new drugs for GIST. The number of drugs in preclinical studies reflects the vitality of research and development of new drugs. Only eight drugs are currently in preclinical studies. This study summarized the therapeutic targets and their frequency in GIST clinical trials of medicines. And it also showed the trend in the number of GIST trials over the years. According to our data, we have not seen a significant growth trend in new drugs for the treatment of GIST.

## Figures and Tables

**Table 1:** Drugs in the GIST field and their development status (>=Phase III Clinical Trial).

**Generic Drug Name**	**Development Status**	**Highest Status Reached**
Imatinib mesilate	Widely Launched	Launched
Sunitinib	Widely Launched	Launched
Regorafenib	Active	Launched
Ripretinib	Active	Launched
Avapritinib	Active	Launched
Masitinib	Ceased	Pre-registration
Bevacizumab	Active	Phase III Clinical Trial
Crenolanib	Active	Phase III Clinical Trial
Famitinib	Active	Phase III Clinical Trial
Nilotinib	Active	Phase III Clinical Trial
Pimitespib	Active	Phase III Clinical Trial
Motesanib diphosphate	Ceased	Phase III Clinical Trial
Retaspimycin	Ceased	Phase III Clinical Trial
Ridaforolimus	Ceased	Phase III Clinical Trial
Trebananib	Ceased	Phase III Clinical Trial

**Table 2:** Targets of launched drugs.

**Generic Drug Name**	**Target**	**Company**
Imatinib mesilate	BCR activator of RhoGEF and GTPase ABL1 PDGFRA KIT	Novartis
Sunitinib	PDGFRA FLT3 KIT RET FLT1 (VEGFR1) KDR (VEGFR2) FLT4 (VEGFR3)	Pfizer
Regorafenib	FLT1 (VEGFR1) KDR (VEGFR2) FLT4 (VEGFR3) KIT RET PDGFRA PDGFR-β B-Raf Raf-1 FGFR1 TIE2	Bayer Amgen
Ripretinib	KIT PDGFRA	Deciphera Pharmaceuticals Zai Lab Specialised Therapeutics
Avapritinib	PDGFRA D842V KIT D816V	Blueprint Medicines CStone Pharmaceuticals

**Table 3:** Targets of drugs in active phase III clinical trials.

**Generic Drug Name**	**Target**	**Company**
Bevacizumab	VEGF	Roche
Crenolanib	PDGFRA PDGFR-β FLT3	Arog Pharmaceuticals JI Shanghai Biotechnology Astellas Pharma Pfizer
Famitinib	KDR (VEGFR2) FLT4 (VEGFR3) KIT FLT3	Jiangsu Hengrui Pharmaceuticals
Nilotinib	BCR activator of RhoGEF and GTPase ABL1 KIT PDGFRA	Novartis
Pimitespib	Hsp90	Otsuka Holdings

**Table 4:** Targets of drugs in active phase II clinical trials.

**Generic Drug Name**	**Target**	**Company**
Amcasertib	Unspecified	1Globe Biomedical Sumitomo Dainippon Pharma
Anlotinib hydrochloric	KDR (VEGFR2) FLT4 (VEGFR3) FGFR1 FGFR2 FGFR3 FGFR4	Sino Biopharmaceutical
Apatinib	KDR (VEGFR2) RET	HLB LifeScience Bukwang Pharmaceutical Jiangsu Hengrui Pharmaceuticals
Bezuclastinib	KIT	Cogent Biosciences Daiichi Sankyo
Dabrafenib	B-Raf	Novartis
Dovitinib lactate	FGFR1 FGFR3 FLT1 (VEGFR1) KDR (VEGFR2) FLT4 PDGFR-β KIT FLT3 EGFR	Allarity Therapeutics Novartis
Everolimus	mTOR	Novartis
Evofosfamide	Unspecified	Molecular Templates
Ilixadencel	Unspecified	Immunicum
Midostaurin	PKC alpha FLT3 KIT VEGF cyclin B1 FLT1 (VEGFR1) KDR (VEGFR2) PDGFR-β	Novartis
Olaratumab	PDGFRA	Bristol-Myers Squibb Eli Lilly
Onalespib	Hsp90	Otsuka Holdings
Pexidartinib	KIT CSF1 receptor FLT3	Daiichi Sankyo
Ponatinib	BCR activator of RhoGEF and GTPase ABL FLT1 (VEGFR1) FGFR 1 FGFR 2 FGFR 3 FGFR 4 FLT3 TEK	Takeda
Trametinib	MEK1 MEK2	Novartis
Temozolomide	Unspecified	Merck Sharp Dohme

**Table 5:** Active drugs in phase I clinical trials and preclinical research.

**Generic Drug Name**	**Development Status**	**Highest Status Reached**
177Lu-NEOBOMB1	Active	Phase I Clinical Trial
Alpelisib	Active	Phase I Clinical Trial
Buparlisib	Active	Phase I Clinical Trial
Cabozantinib	Active	Phase I Clinical Trial
Copanlisib	Active	Phase I Clinical Trial
Dasatinib	Active	Phase I Clinical Trial
DS-6157	Active	Phase I Clinical Trial
Lenalidomide	Active	Phase I Clinical Trial
Pazopanib	Active	Phase I Clinical Trial
Pegargiminase	Active	Phase I Clinical Trial
Plinabulin	Active	Phase I Clinical Trial
Quizartinib dihydrochloride	Active	Phase I Clinical Trial
Sapacitabine	Active	Phase I Clinical Trial
Surufatinib	Active	Phase I Clinical Trial
Tidutamab	Active	Phase I Clinical Trial
Umbralisib	Active	Phase I Clinical Trial
Anagrelide, Sartar Therapeutics	Active	Preclinical
Cediranib	Active	Preclinical
GT-1708F	Active	Preclinical
IM-24	Active	Preclinical
NN-3201	Active	Preclinical
Plocabulin	Active	Preclinical
Sugemalimab	Active	Preclinical
THE-630	Active	Preclinical

## Data Availability

The data that support the findings of this study are available at https://doi.org/10.6084/m9.figshare.21258447.v1.

## LIST OF ABBREVIATIONS

EGFR: Epidermal Growth Factor Receptor
FGFR: Fibroblast Growth Factor Receptor
FLT: Fms-Related Tyrosine Kinase
GIST: Gastrointestinal Stromal Tumors
HAP: Hypoxia-Activated Prodrug
KDR: Kinase Insert Domain Receptor
MEK: Mitogen-Activated Protein Kinase Kinase
mTOR: Mechanistic Target of Rapamycin Kinase
PDGFR-β: Platelet Derived Growth Factor Receptors Beta
PDGFRA: Platelet Derived Growth Factor Receptors Alpha
PKC-alpha: Protein Kinase C alpha
RET: Glial Cell-Line Derived Neurotrophic Factor Receptor
SCF: Stem-Cell Factor
TKIs: Tyrosine Kinase Inhibitors
VEGF: Vascular Endothelial Growth Factor A
VEGFR: Vascular Endothelial Growth Factor Receptors
