# Supplementary material for: Drugs in the GIST Field (Therapeutic Targets and Clinical Trial Staging)
Source: Curr Drug Deliv. 2023 Sep 28;21(1):80–90. doi: 10.2174/1567201820666221122120657 (PMC10661963; doi:10.2174/1567201820666221122120657)
Supplement: Supplementary file 1 — Supplementary material is available on the publisher’s website along with the published article. [file CDD-21-80_SD1.pdf]

SUPPLEMENTARY MATERIAL

Drugs In The GIST Field (Therapeutic Targets and Clinical Trial Staging)

Chen Huang[1,#], Xinli Ma[1,#], Ming Wang[1] and Hui Cao[1,*]

[1]*Department of Gastrointestinal Surgery, Renji Hospital, School of Medicine, Shanghai Jiao Tong University, Shanghai, China*

Supplementary Table 1. The full list of drugs in the GIST field and their development status.

Generic Drug Name	Development Status	Highest Status Reached
imatinib mesilate	Widely Launched	Launched
sunitinib	Widely Launched	Launched
regorafenib	Active	Launched
ripretinib	Active	Launched
avapritinib	Active	Launched
masitinib	Ceased	Pre-registration
bevacizumab	Active	Phase III Clinical Trial
crenolanib	Active	Phase III Clinical Trial
famitinib	Active	Phase III Clinical Trial
nilotinib	Active	Phase III Clinical Trial
pimitespib	Active	Phase III Clinical Trial
motesanib diphosphate	Ceased	Phase III Clinical Trial
retaspimycin	Ceased	Phase III Clinical Trial
ridaforolimus	Ceased	Phase III Clinical Trial
trebananib	Ceased	Phase III Clinical Trial
amcasertib	Active	Phase II Clinical Trial
anlotinib hydrochloric	Active	Phase II Clinical Trial
apatinib	Active	Phase II Clinical Trial<br>Phase I Clinical Trial<br>Phase II Clinical Trial
bezuclastinib	Active	Phase II Clinical Trial
dabrafenib	Active	Phase II Clinical Trial
dovitinib lactate	Active	Phase II Clinical Trial<br>Phase II Clinical Trial
everolimus	Active	Phase II Clinical Trial
evofosfamide	Active	Phase II Clinical Trial
ilixadencel	Active	Phase II Clinical Trial
midostaurin	Active	Phase II Clinical Trial

olaratumab	Active	Phase II Clinical Trial
onalespib	Active	Phase II Clinical Trial
pexidartinib	Active	Phase II Clinical Trial
ponatinib	Active	Phase II Clinical Trial
trametinib	Active	Phase II Clinical Trial
temozolomide	Active	Phase II Clinical Trial
amonafide dihydrochloride	Ceased	Phase II Clinical Trial
amuvatinib hydrochloride	Ceased	Phase II Clinical Trial
CNF-2024	Ceased	Phase II Clinical Trial
ganetespib	Ceased	Phase II Clinical Trial
NRC-AN-019	Ceased	Phase II Clinical Trial
SMi-BX1	Ceased	Phase II Clinical Trial
177Lu-NEOBOMB1	Active	Phase I Clinical Trial
alpelisib	Active	Phase I Clinical Trial
buparlisib	Active	Phase I Clinical Trial
cabozantinib	Active	Phase I Clinical Trial
copanlisib	Active	Phase I Clinical Trial
dasatinib	Active	Phase I Clinical Trial
DS-6157	Active	Phase I Clinical Trial
lenalidomide	Active	Phase I Clinical Trial
pazopanib	Active	Phase I Clinical Trial
pegargiminase	Active	Phase I Clinical Trial
plinabulin	Active	Phase I Clinical Trial
quizartinib dihydrochloride	Active	Phase I Clinical Trial
sapacitabine	Active	Phase I Clinical Trial
surufatinib	Active	Phase I Clinical Trial
tidutamab	Active	Phase I Clinical Trial
umbralisib	Active	Phase I Clinical Trial
KTN-0158	Ceased	Phase I Clinical Trial
LOP-628	Ceased	Phase I Clinical Trial
MK-1496	Ceased	Phase I Clinical Trial
OPB-51602	Ceased	Phase I Clinical Trial
perifosine	Ceased	Phase I Clinical Trial
refametinib	Ceased	Phase I Clinical Trial
XL-820	Ceased	Phase I Clinical Trial
anagrelide, Sartar Therapeutics	Active	Preclinical
cediranib	Active	Preclinical

GT-1708F	Active	Preclinical
IM-24	Active	Preclinical
NN-3201	Active	Preclinical
plocabulin	Active	Preclinical
sugemalimab	Active	Preclinical
THE-630	Active	Preclinical
AB-515	Ceased	Preclinical
AZD-3229	Ceased	Preclinical
c-kit inhibitors, Deciphera	Ceased	Preclinical
gastrointestinal stromal tumour therapy, Array BioPharma	Ceased	Preclinical
HYGT-110	Ceased	Preclinical
LWEL-1808	Ceased	Preclinical
MAAC-003	Ceased	Preclinical

Supplementary Table 2. Therapeutic targets and production companies of ceased drugs.

Generic Drug Name	Company	Development Status	Highest Status Reached	Target
masitinib	AB Science	Ceased	Pre-registration	platelet derived growth factor receptor beta<br>KIT proto-oncogene, receptor tyrosine kinase<br>fibroblast growth factor receptor 3<br>LYN proto-oncogene, Src family tyrosine kinase<br>FYN proto-oncogene, Src family tyrosine kinase<br>colony stimulating factor 1 receptor
motesanib diphosphate	Amgen<br>Takeda	Ceased	Phase III Clinical Trial	fms related tyrosine kinase 1<br>kinase insert domain receptor<br>fms related tyrosine kinase 4<br>KIT proto-oncogene, receptor tyrosine kinase<br>platelet derived growth factor receptor alpha<br>ret proto-oncogene
retaspimycin	AstraZeneca<br>Infinity Pharmaceuticals	Ceased	Phase III Clinical Trial	heat shock protein 90 alpha family class A member 1
ridaforolimus	Takeda<br>Merck & Co.	Ceased	Phase III Clinical Trial	mechanistic target of rapamycin kinase
trebananib	Amgen<br>Takeda	Ceased	Phase III Clinical Trial	TEK receptor tyrosine kinase<br>angiopoietin 2<br>angiopoietin 1
amonafide dihydrochloride	Teva	Ceased	Phase II Clinical Trial	DNA topoisomerase II alpha
amuvatinib hydrochloride	Otsuka Holdings	Ceased	Phase II Clinical Trial	RAD51 recombinase<br>KIT proto-oncogene, receptor tyrosine kinase<br>platelet derived growth factor receptor alpha<br>fms related tyrosine kinase 3<br>MET proto-oncogene, receptor tyrosine kinase
CNF-2024	Biogen	Ceased	Phase II Clinical Trial	heat shock protein 90 alpha family class A member 1

ganetespib	Aldeyra Therapeutics<br>Ergomed<br>Madrigal Pharmaceuticals	Ceased	Phase II Clinical Trial	heat shock protein 90 alpha family class A member 1<br>WT1 transcription factor
NRC-AN-019	Natco Pharma	Ceased	Phase II Clinical Trial	Unspecified
SMi-BX1	CTI BioPharma	Ceased	Phase II Clinical Trial	Unspecified
KTN-0158	Celldex Therapeutics	Ceased	Phase I Clinical Trial	KIT proto-oncogene, receptor tyrosine kinase
LOP-628	ImmunoGen<br>Novartis	Ceased	Phase I Clinical Trial	platelet derived growth factor receptor alpha
MK-1496	Merck & Co.	Ceased	Phase I Clinical Trial	polo like kinase 1
OPB-51602	Otsuka Holdings	Ceased	Phase I Clinical Trial	signal transducer and activator of transcription 3
perifosine	Handok<br>Hikma Pharmaceuticals<br>Yakult Honsha<br>Aeterna Zentaris<br>Akebia Therapeutics<br>Nippon Kayaku<br>Viatris	Ceased	Phase I Clinical Trial	AKT serine/threonine kinase 1<br>phosphatidylinositol-4,5-bisphosphate 3-kinase catalytic subunit alpha
refametinib	AstraZeneca<br>AstraZeneca<br>Bayer<br>Bayer<br>Bayer	Ceased	Phase I Clinical Trial	mitogen-activated protein kinase kinase 1<br>mitogen-activated protein kinase kinase 2
XL-820	Exelixis	Ceased	Phase I Clinical Trial	KIT proto-oncogene, receptor tyrosine kinase<br>platelet derived growth factor receptor alpha<br>kinase insert domain receptor
AB-515	Daiichi Sankyo	Ceased	Preclinical	ABL proto-oncogene 1, non-receptor tyrosine kinase<br>KIT proto-oncogene, receptor tyrosine kinase
AZD-3229	AstraZeneca<br>Ningbo Tai Kang Medical Tech	Ceased	Preclinical	KIT proto-oncogene, receptor tyrosine kinase<br>platelet derived growth factor receptor alpha
c-kit inhibitors, Deciphera	Deciphera Pharmaceuticals	Ceased	Preclinical	KIT proto-oncogene, receptor tyrosine kinase<br>kinase insert domain receptor<br>platelet derived growth factor receptor alpha
gastrointestinal stromal tumour therapy, Array Bio-Pharma	Clovis Oncology<br>Pfizer	Ceased	Preclinical	KIT proto-oncogene, receptor tyrosine kinase
HYGT-110	Hefei Cosource Pharmaceu-tical	Ceased	Preclinical	Unspecified
LWEL-1808	Luckwel Pharmaceuticals	Ceased	Preclinical	Unspecified
MAAC-003	MAA Laboratories	Ceased	Preclinical	Unspecified

Supplementary Table 3. Frequency of all therapeutic targets in GIST clinical trials of medicines.

Generic Drug Name	Frequency
KIT	25
PDGFRA	16
KDR/VEGFR2	13
FLT3	10
FLT1/VEGFR1	8
FLT4/VEGFR3	8
ABL	5
RET	5
PDGFRB	5
FGFR3	5
Hsp90	5
FGFR1	5
BCR activator of RhoGEF and GTPase	4
PIK3CA	4
B-Raf	3
CSF1 receptor	3
TEK	3
PI3K p110δ	3
LYN	2
FYN	2
VEGF	2
mTOR	2
MET	2
FGFR2	2
FGFR4	2
MEK1	2
MEK2	2
PIK3CB	2
PIK3CG	2
Raf-1	1
Angpt2	1
Angpt1	1
TOP2A	1
RAD51 recombinase	1

GRPR	1
EGFR	1
WT1	1
PKC-alpha	1
cyclin B1	1
AXL	1
SRC	1
YES	1
LCK	1
GPCR-20	1
Top1	1
cereblon	1
PLK1	1
STAT3	1
AKT1	1
Rho/Rac guanine nucleotide exchange factor 2	1
DNA polymerase alpha 1	1
SSTR2	1
CD3e	1
CSNK1E	1
PDE3A	1
SMO	1
CD274 molecule	1
